# Erratum

**DOI:** 10.1093/ofid/ofx181

**Published:** 2018-01-30

“Point-of-Care Viral Load Testing for Sub-Saharan Africa: Informing a Target Product Profile” by Phillips et al. [Open Forum Infect Dis **2016**; 3(3)] is an Open Access article and should have contained the following copyright line:

© The Author [2018]. Published by Oxford University Press [on behalf of the Infectious Diseases Society of America]. This is an Open Access article distributed under the terms of the Creative Commons Attribution License (http://creativecommons.org/licenses/by/4.0/), which permits unrestricted reuse, distribution, and reproduction in any medium, provided the original work is properly cited.

The authors regret these error.

